# Orthopedic Surgery Residency Program Website Content and Accessibility During the COVID-19 Pandemic: Observational Study

**DOI:** 10.2196/30821

**Published:** 2021-09-10

**Authors:** Muhammad El Shatanofy, Lauryn Brown, Peter Berger, Alex Gu, Abhinav K Sharma, Joshua Campbell, Sean Tabaie

**Affiliations:** 1 Department of Orthopedic Surgery The George Washington University School of Medicine and Health Sciences Washington, DC United States; 2 Department of Orthopedic Surgery School of Medicine University of California, Irvine Orange, CA United States; 3 Division of Orthopedics and Sports Medicine Children’s National Medical Center Washington, DC United States

**Keywords:** orthopedic surgery residency programs, COVID-19, website, residency applicants, residency, medical student, content, accessibility, observational, surgery

## Abstract

**Background:**

The exceptional competitiveness of the orthopedic surgery specialty, combined with the unclear impact of the COVID-19 pandemic on residency recruitment, has presented significant challenges to applicants and residency program directors. With limited in-person opportunities in the 2020-2021 application cycle, applicants have been pressed to gauge chances and best fit by browsing program websites.

**Objective:**

The aim of the study was to assess the accessibility and content of accredited orthopedic surgery residency program websites during the COVID-19 pandemic.

**Methods:**

Using the online database of the Electronic Residency Application Service (ERAS), we compiled a list of accredited orthopedic surgery residency programs in the United States. Program websites were evaluated across four domains: program overview, education, research opportunities, and application details. Each website was assessed twice in July 2020, during a period of adjustment to the COVID-19 pandemic, and twice in November 2020, following the October ERAS application deadline.

**Results:**

A total of 189 accredited orthopedic surgery residency programs were identified through ERAS. Of these programs, 3 (1.6%) did not have functional website links on ERAS. Data analysis of content in each domain revealed that most websites included program details, a description of the didactic curriculum, and sample rotation schedules. Between the two evaluation periods in July and November 2020, the percentage of program websites containing informative videos and virtual tours rose from 12.2% (23/189) to 48.1% (91/189; *P*<.001) and from 0.5% (1/189) to 13.2% (25/189; *P*<.001), respectively. However, the number of programs that included information about a virtual subinternship or virtual interview on their websites did not change. Over the 4-month period, larger residency programs with 5 or more residents were significantly more likely to add a program video (*P*<.001) or virtual tour (*P*<.001) to their websites.

**Conclusions:**

Most residency program websites offered program details and an overview of educational and research opportunities; however, few addressed the virtual transition of interviews and subinternships during the COVID-19 pandemic.

## Introduction

Applicants to orthopedic surgery residencies spend significant time and resources gathering information about potential programs [1-7]. A valuable resource that has been shown to influence application decisions across specialties is program websites [8]. Multiple studies have found that applicants heavily use residency websites when deciding where to apply and interview and, subsequently, how to rank programs [7,9-11]. In orthopedics specifically, Yong et al surveyed 610 applicants to an orthopedic surgery residency program and found that 98% of students used program websites to gather information [9].

Despite the clear utility of websites for residency applicants and programs alike, content is inconsistent and often severely lacking. In a recent review of orthopedic surgery residency websites, Oladeji et al found widespread inconsistencies and noted a scarcity of information desired by prospective applicants [12]. Yong et al also found that, although applicants referenced websites frequently, the quality of information was ranked lower than that provided by medical school advisors or orthopedic surgery residents at home programs [9]. While it was possible to supplement inadequate information found on websites with in-person experiences in previous years, the COVID-19 pandemic has limited this year’s applicants to mostly virtual experiences. Consequently, applicants have been forced to rely more heavily on websites in the 2020-2021 application cycle [9,13-15].

The purpose of this study was to assess the content and quality of orthopedic surgery residency program websites and to evaluate adaptations made in response to the COVID-19 pandemic. We hypothesized that, despite the evolving pandemic, updates to program websites, videos, and virtual tours would be limited. This study aims to both describe how orthopedic surgery programs adapted their websites in light of unprecedented circumstances and provide actionable items for programs to improve their online presence during future application cycles.

## Methods

### Overview

The Electronic Residency Application Service (ERAS) website contains an updated list of all accredited orthopedic surgery residency programs in the United States [16]. Upon accessing this website in July 2020, 189 accredited programs were identified and included in this study. A hyperlink to each program’s website and accreditation IDs were gathered from the ERAS website. The hyperlinks were then accessed and classified as functional, indirect (ie, functional link, but required navigation to reach the orthopedic surgery residency page), or direct. Two authors independently gathered information from each website at two separate time periods of the application cycle. The first data collection occurred in the first 2 weeks of July 2020, a period of relative adjustment to COVID-19, and the second data collection occurred within the first 2 weeks of November 2020, a period shortly after the ERAS application deadline.

Information gathered from the orthopedic surgery websites fell into four broad domains: program overview, education, research opportunities, and application details.

### Program Overview

Program overview included program director name; contact information, including email, phone number, and address; fellowship match lists; wellness opportunities; and salary and benefits information. Efforts to promote diversity were also reported for programs that mentioned underrepresented minorities or gender diversity in their mission statements.

### Education

The education domain included the mention of extracurricular meetings and courses (ie, travel to conferences), didactic sessions, a journal club, sample rotation schedules, clinic and call responsibilities, and educational support, such as funding for loupes and leads.

### Research

Research opportunities were identified by scanning websites for evidence of a research requirement, publication lists, lab spaces, or funding for national presentations and conferences.

### Application Details

Application details gathered from the websites included the number of residents accepted into each program per year, a subinternship description, and guidelines for United States Medical Licensing Examination (USMLE) Step 2 score and Electronic Standardized Letter of Recommendation (eSLOR) submission.

### Virtual Updates

Due to the nature of this virtual application cycle, websites were also assessed for the inclusion of program videos, video lengths if applicable, virtual tours, remote opportunities such as virtual subinternships, and details about virtual interviews.

All data collected from this study were analyzed after the second website review in November 2020. Data from July 2020 were compared to data from November 2020 to assess how programs have modified their websites in response to the virtual application cycle. Unless otherwise noted, statistics were reported on data obtained in July 2020. Analyses were performed using paired-sample *t* tests, Pearson chi-square tests, and Fisher exact tests. Significance was established at a *P* value of .05.

## Results

### Program Overview

Overall, 189 residency programs were identified on ERAS in July 2020. All but 3 residency programs (n=186, 98.4%) listed a functional link to the program website. Most programs listed the program director’s name in July 2020 (n=164, 86.8%; Table 1). Email and phone number were included in 85.7% (n=162) and 84.1% (n=159) of websites, respectively, while address was included in 65.1% (n=123) of websites. Only 22.2% (n=42) of websites addressed underrepresented minorities and 20.6% (n=39) mentioned gender diversity. Efforts to promote wellness or engagement in social events were identified among 50.3% (n=95) of websites. Only 56.1% (n=106) of programs included a fellowship match list. Benefits and salary information was included in 63.5% (n=120) of websites.

**Table 1:** Content of orthopedic surgery residency program websites in July 2020.

Category			Value (N=189)
**Program overview, n (%)**			
	Program director	164 (86.8)	
	Address	123 (65.1)	
	Phone	159 (84.1)	
	Email	162 (85.7)	
	Address underrepresented minorities	42 (22.2)	
	Address gender diversity	39 (20.6)	
	Wellness	95 (50.3)	
	Fellowship match list	106 (56.1)	
	Salary and benefits	120 (63.5)	
**Education, n (%)**			
	Didactics	159 (84.1)	
	Journal club	129 (68.3)	
	Rotation schedule	132 (69.8)	
	Call responsibility	96 (50.8)	
	Educational support	92 (48.7)	
	Meetings and courses	106 (56.1)	
	International opportunities	32 (16.9)	
**Research, n (%)**			
	Research requirement	138 (73.0)	
	Research output	68 (36.0)	
	Research support	139 (73.5)	
**Application details, n (%)**			
	Electronic Standardized Letter of Recommendation	27 (14.3)	
	Step 2	53 (28.0)	
Number of residents per year, mean (SD)			4.6 (2.1)

### Education

Of the 189 program websites, 84.1% (n=159) noted didactic sessions, 68.3% (n=129) mentioned a journal club, and 69.8% (n=132) included a sample rotation schedule (Table 1). Less commonly reported metrics included mention of meetings and courses outside of the traditional program curriculum (n=106, 56.1%), call responsibilities (n=96, 50.8%), international opportunities (n=32, 16.9%), and educational support (n=92, 48.7%).

### Research

Most of the 189 program websites noted a research requirement (n=138, 73.0%) and demonstrated research support (n=139, 73.5%), such as funding for residents (Table 1). Research output, such as a list of resident publications, was less commonly included among websites (n=68, 36.0%).

### Application Details

The average number of residents accepted into each program ranged from 4 to 5 residents per year (SD 2.1). Upon reviewing application requirements, only 28.0% (n=53) of 189 websites mentioned a Step 2 score requirement and 14.3% (n=27) requested an eSLOR. 

### Virtual Updates

**Figure 1:**
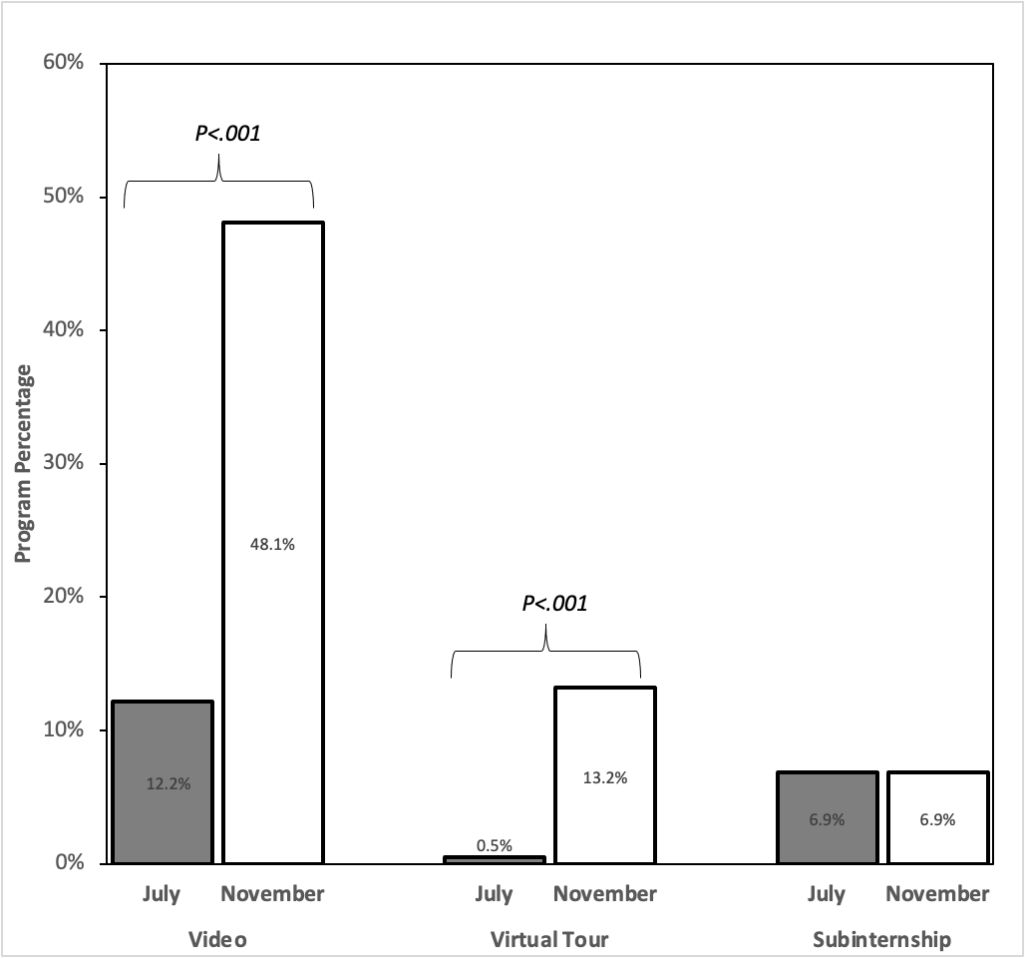
Percentage of orthopedic surgery residency program websites with updated virtual information between July and November 2020.

Between July and November 2020, the number of program websites out of 189 that mentioned a virtual subinternship experience remained unchanged at 6.9% (n=13; *P*>.99; Figure 1). The percentage of websites including a program video rose from 12.2% (n=23) to 48.1% (n=91; *P<*.001), and the percentage of websites including a virtual tour increased from 0.5% (n=1) to 13.2% (n=25; *P<*.001; Table 2). A total of 71 program videos were identified across all 186 programs with functional websites in November 2020. The length of the videos ranged from 57 seconds to 24 minutes and 40 seconds.

A chi-square analysis was performed to gauge whether larger programs, characterized as having 5 or more residents per year, were more likely than smaller programs to add program videos or virtual tours by November 2020 (Table 3). Of the 94 larger programs, 48% (n=45) added videos, compared to only 24% (n=23) of the 95 smaller programs (*P*<.001; Multimedia Appendix 1). Larger programs (20/94, 21%) were also more likely than smaller programs (4/95, 4%) to add virtual tours by November 2020 (*P*<.001; Multimedia Appendix 2).

**Table 2:** Virtual offerings identified on program websites in July and November 2020.

Category	Websites (N=189), n (%)			*P* value^a^
	July 2020	November 2020		
Program video	23 (12.2)	91 (48.1)	<.001	
Virtual tour	1 (0.5)	25 (13.2)	<.001	
Subinternship	13 (6.9)	13 (6.9)	>.99	

^a^Significance was established at *P*<.05.

**Table 3:** Orthopedic residency program websites with added virtual content between July and November 2020 compared by program size.

Category	Websites, n (%)			*P* value^a^
	Small programs (<5 residents/year) (n=95)	Large programs (≥5 residents/year) (n=94)		
Added program video	23 (24)	45 (48)	<.001	
Added virtual tour	4 (4)	20 (21)	<.001	

^a^Significance was established at *P*<.05.

## Discussion

### Principal Findings

The exceptional competitiveness of the orthopedic surgery specialty, compounded with the unclear impact of COVID-19 on residency recruitment, has presented significant challenges to both applicants and programs. Since most in-person opportunities and interviews in the 2020-2021 match cycle were cancelled due to COVID-19, we anticipated that applicants would increasingly rely on residency websites to gain insight into programs and cultural fit [13,17-19]. The purpose of this study was to explore the extent to which orthopedic surgery residency websites were updated throughout the pandemic. 

In our study, we accounted for 98.4% (n=186) of the existing 189 orthopedic surgery residency programs through either hyperlinks provided by ERAS or via Google search. We reasoned that the programs would prioritize making COVID-19–related adjustments to websites prior to the ERAS application deadline in October 2020. Therefore, we recorded data in July 2020 and again in November 2020, once the ERAS deadline expired. Our analyses mostly supported our original hypothesis. While the percentage of program videos rose significantly from 12.2% (23/189) to 48.1% (91/189; *P*<.001) and the percentage of virtual tours rose significantly from 0.5% (1/189) to 13.2% (25/189; *P*<.001) from July to November 2020, the percentage of websites that mentioned a virtual subinternship experience remained stagnant at 6.9% (13/189). This is concerning because, historically, the role of the subinternship in pursuing orthopedic surgery residencies has been to provide both visiting students and programs an opportunity to assess fit based on personal skills, clinical aptitude, and the ability to integrate into program culture [5].

While our results suggest applicants would struggle to find updated information on websites regardless of program characteristics, virtual offerings were also evaluated by program size. Large programs with 5 or more residents were significantly more likely to add a program video (*P*<.001) and a virtual tour (*P*<.001) to their websites between July and November 2020. Further, with less than half (45/94, 48%) of larger residency programs and less than a quarter (23/95, 24%) of smaller programs adding a program video during the application season, applicants have been tasked with learning more about orthopedic surgery residency programs using dated videos and online information. Additionally, with only 21% (20/94) of larger programs and 4% (4/95) of smaller programs adding a virtual tour, applicants have limited representations of the physical environment surrounding their potential residency placements. Collectively, these findings indicate that smaller programs were at a potential disadvantage for recruiting applicants since they were less likely than larger programs to have information that applicants would find critical in lieu of in-person interaction.

### Comparison With Prior Work

Consistent with previous research, this study identified gaps in the quantity and quality of information on orthopedic surgery residency websites. Rozental et al completed the first review of orthopedic program websites at a time when only 40% of the United States had access to the internet and discovered that only 113 of 154 programs (73.4%) had working websites [20]. In a follow-up study conducted by Oladeji et al, 97% of programs had websites, but less than 50% provided information about call schedules, resident benefits, and resident research [12]. All of these factors have been ranked as important to residents [7,21]. Between the shared categories with Oladeji et al, we found that more programs mentioned resident salary, resident research requirements, publications, research and educational support, journal clubs, and didactics [12]. Information on call responsibility rose slightly to 50.8% (96/189), and resident wellness activities remained at 50.3% (95/189). Only information regarding rotation schedules dropped between studies (132/189, 69.8%).

For data collection unique to our study, we found that international opportunities were listed on 16.9% (32/189) of websites and fellowship match lists were included on 56.1% (106/189) of websites. While the mention of international opportunities was scarce, the low percentage of fellowship match lists was particularly concerning, given that over 90% of residents choose to complete an orthopedic surgery fellowship following graduation [22]. We also found that, despite the anticipated changes to application metrics, including scoring changes to USMLE Step 1, only 28.0% (53/189) of websites mentioned USMLE Step 2 application requirements and 14.3% (27/189) indicated preferences for an eSLOR [23].

Perhaps the most concerning of our findings was the low effort to promote racial and gender diversity on websites. Less than 25% of programs addressed either underrepresented minorities (42/189, 22.2%) or gender diversity (39/189, 20.6%). Over the past 10 years, racial and gender diversity among orthopedic surgery residency programs has remained stagnant compared to the rise observed in medical schools [24]. Despite feedback provided by faculty and residents, orthopedic surgery residency programs continue to have the lowest ratio of female to male residents than any other specialty [19,24].

### Limitations

Several limitations to this study exist. Since this study started in July 2020, we could not capture COVID-19–related changes prior to this period. Additionally, authors only documented if variables were mentioned on the websites and did not assess the quality of the information. Although data collection by two authors added to the internal validity of the study, we could not control for interrater variability. This study also did not include potential items of interest, such as interview dates, cases performed and their volumes, and operative approaches.

### Recommendations

The data for this study were collected within a 5-month period from July to November 2020. Although traffic metrics are unavailable, it is reasonable to assume that most applicants visited sites during this time to prepare for ERAS deadlines. This study identified an overall paucity of information on program sites and an inadequate response to the COVID-19 pandemic. Further, websites were difficult to navigate, and important information was dispersed across several tabs. This may have led applicants to overlook time-sensitive application requirements and miss deadlines.

We propose several recommendations to improve website quality and quantity of information during the current pandemic and future states of emergency. First, we encourage the ERAS directory of programs to include hyperlinks for all orthopedic surgery residency programs. If a functional hyperlink to a program cannot be found, ERAS should contact the program and encourage it to either provide a link or create a new one if unavailable. Second, all programs should be made aware of standardized information and organization that applicants find useful, such as the ones described in this study. It will ultimately be left to the discretion of the programs whether or not to adjust.

Due to the evolving situation of the COVID-19 pandemic, ERAS should encourage and potentially require websites to upload monthly updates. Uploading information about the program’s response to COVID-19 not only has implications for recruitment, but also addresses concerns about safety [8,25]. Since travel restrictions have limited physical visits by applicants to programs, programs should also be encouraged to include at least one virtual tour and one program video on their websites. Additional videos should be uploaded to highlight program diversity and wellness. To make the application process more personable, applicants should also have the option to schedule video meetings with current residents and faculty via program websites [26]. The COVID-19 pandemic has drastically altered the virtual arena. Making the changes proposed in this study will undeniably facilitate the application process for future residents.

### Conclusions

This study highlights the inadequate response of orthopedic surgery residency programs to update their websites during this entirely virtual application cycle. As a competitive specialty with the third-lowest specialty match rate, orthopedic surgery programs still have a lot of work to do to improve their online presence, promote diversity, and enhance opportunities for virtual applicants [1,19]. With limited information, applicants must identify unique ways to learn about residency programs and gauge their chances for a successful match.

## Appendix

Multimedia Appendix 1 Orthopedic surgery residency program websites with added videos between July and November 2020 compared by program size.

Multimedia Appendix 2 Orthopedic surgery residency program websites with added virtual tours between July and November 2020 compared by program size.

## Abbreviations

ERAS: Electronic Residency Application Service
eSLOR: Electronic Standardized Letter of Recommendation
USMLE: United States Medical Licensing Examination
