# Supplementary figures and images for: Orthopedic Surgery Residency Program Website Content and Accessibility During the COVID-19 Pandemic: Observational Study
Source: JMIR Med Educ. 2021 Sep 10;7(3):e30821. doi: 10.2196/30821 (PMC8437405; doi:10.2196/30821)

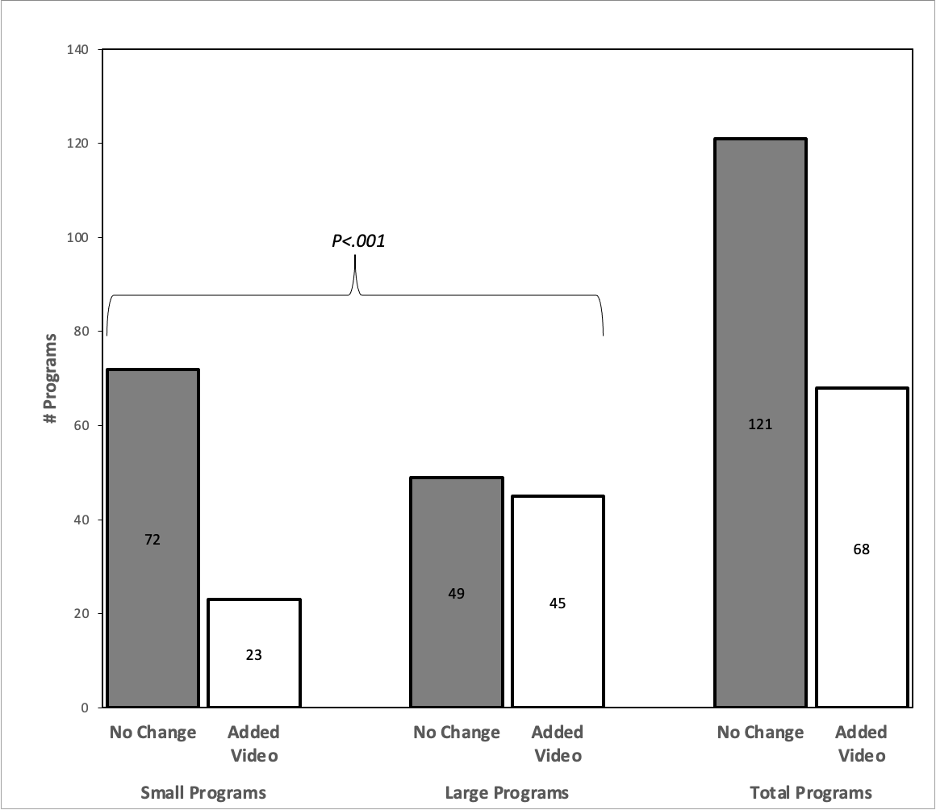

Supplement: Multimedia Appendix 1 [file mededu_v7i3e30821_app1.png]

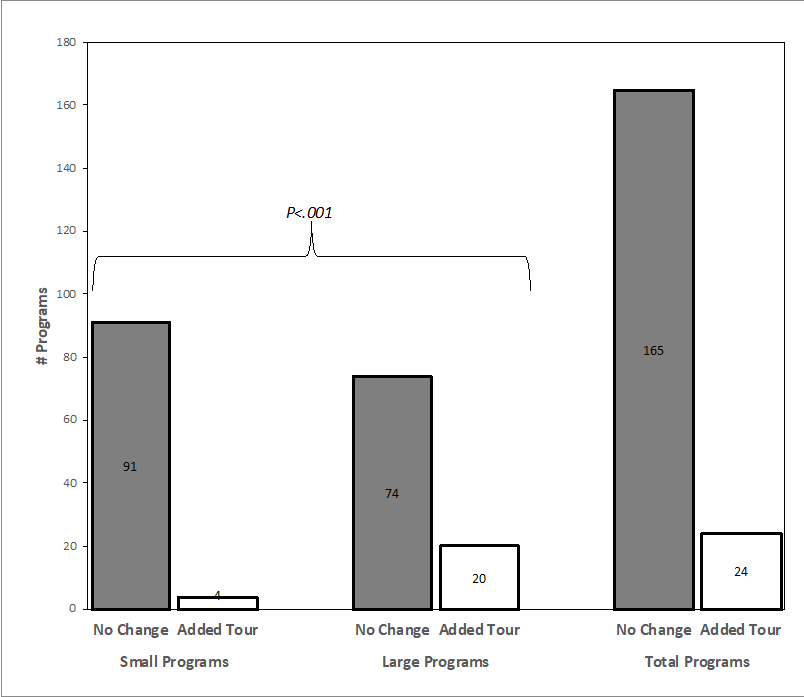

Supplement: Multimedia Appendix 2 [file mededu_v7i3e30821_app2.png]
